# Puzzling Findings in Studying the Outcome of “Real World” Adolescent Mental Health Services: The TRAILS Study

**DOI:** 10.1371/journal.pone.0044704

**Published:** 2012-09-19

**Authors:** Frederike Jörg, Johan Ormel, Sijmen A. Reijneveld, Daniëlle E. M. C. Jansen, Frank C. Verhulst, Albertine J. Oldehinkel

**Affiliations:** 1 Interdisciplinary Centre Psychopathology and Emotion regulation, University Medical Centre Groningen, University of Groningen, Groningen, The Netherlands; 2 Department of Health Sciences, University Medical Centre Groningen, University of Groningen, Groningen, The Netherlands; 3 Erasmus University Medical Centre, Sophia Children's Hospital Rotterdam, Rotterdam, The Netherlands; The University of Queensland, Australia

## Abstract

**Background:**

The increased use and costs of specialist child and adolescent mental health services (MHS) urge us to assess the effectiveness of these services. The aim of this paper is to compare the course of emotional and behavioural problems in adolescents with and without MHS use in a naturalistic setting.

**Method and Findings:**

Participants are 2230 (pre)adolescents that enrolled in a prospective cohort study, the TRacking Adolescents' Individual Lives Survey (TRAILS). Response rate was 76%, mean age at baseline 11.09 (SD 0.56), 50.8% girls. We used data from the first three assessment waves, covering a six year period. Multiple linear regression analysis, propensity score matching, and data validation were used to compare the course of emotional and behavioural problems of adolescents with and without MHS use. The association between MHS and follow-up problem score (β 0.20, SE 0.03, p-value<0.001) was not confounded by baseline severity, markers of adolescent vulnerability or resilience nor stressful life events. The propensity score matching strategy revealed that follow-up problem scores of non-MHS-users decreased while the problem scores of MHS users remained high. When taking into account future MHS (non)use, it appeared that problem scores decreased with limited MHS use, albeit not as much as without any MHS use, and that problem scores with continuous MHS use remained high. Data validation showed that using a different outcome measure, multiple assessment waves and multiple imputation of missing values did not alter the results. A limitation of the study is that, although we know what type of MHS participants used, and during which period, we lack information on the duration of the treatment.

**Conclusions:**

The benefits of MHS are questionable. Replication studies should reveal whether a critical examination of everyday care is necessary or an artefact is responsible for these results.

## Introduction

Adolescence is a period in which many boys and girls suffer from emotional and behavioural problems, without these problems always posing a long-term health threat [1]. When problems are severe or persistent, however, mental health care may be indicated. Help seeking behaviour is determined by, among other things, whether or not adolescents or their parents perceive the problems as significant and in need of professional help [2]. At preadolescence, the pathway to care relies on parents' recognition of problems [3]. As adolescents mature, the pathway becomes less certain. Adolescents are probably better informants of their problems [4], but seem less inclined to seek professional help [5].

The increased use and accompanying costs of specialist child and adolescents mental health services (MHS) have heightened the importance of assessing efficacy and cost-effectiveness of services [6]. RCTs in the field typically test one specific intervention in a small, homogeneous sample without complex or co-morbid problems, limiting external validity [7]. There is some evidence from studies comparing care as usual to evidence based treatments [8], [9] that points towards negligible effectiveness of care as usual. Similar results were found in a one-year follow-up study comparing referred to non-referred children [10].

However, studies on outcomes of services provided in a naturalistic setting often suffer from methodological flaws, such as the absence of randomisation, the inclusion of different services and the presence of possible confounding factors [10]. These methodological shortcomings may be dealt with by using sophisticated statistical methods such as propensity score matching, multiple measurement waves, and outcomes on various domains. Using these strategies, we studied the course of emotional and behavioural problems in a population-based cohort of 2230 (pre)adolescents who used or did not use MHS.

The study is carried out in the Netherlands, where mental health care is organised in echelons. For MHS services a referral from the general practitioner (GP) is needed. In the Dutch health care system, everybody is insured for both GP and MHS services; there is no fee for service. The term MHS services includes all inpatient, outpatient and community mental health and social care services for children and adolescents.

## Methods

### Ethics Statement

The study was approved by the Dutch Central Committee on Research Involving Human Subjects. Written informed consent was obtained of all adolescents and their parents after the nature of the study had been fully explained.

### Participants

This study is part of the TRacking Adolescents' Individual Lives Survey (TRAILS), a prospective cohort study of Dutch preadolescents with the aim to explain the development of mental health from preadolescence into adulthood [11]. The present study involves data from the first, second and third assessment wave of TRAILS, which ran from March 2001 to July 2002 (T1), September 2003 to December 2004 (T2), and September 2005 to August 2008 (T3), respectively. TRAILS participants were selected from five municipalities in the North of the Netherlands, including both urban and rural areas. Children born between 1 October 1989 and 30 September 1991 were eligible for inclusion (N = 3483), providing that their schools were willing to cooperate and that they met the inclusion criteria. Over 90% of the schools accommodating 2935 eligible children agreed to participate in the study. 76.0% of these children (N = 2230, mean age = 11.09 years, SD = 0.56, 50.8% girls) were enrolled in the study (i.e., both child and parent agreed to participate). Teacher reports were available for 40.7% of the non-responders, and revealed that they did not differ from responders with respect to the prevalence of problem behaviour, nor regarding associations between socio-demographic variables and mental health outcomes, but were more likely to be boys, have a low socioeconomic background, and perform poorly at school [12]. Of the 2230 T1 participants, 96.4% (N = 2149, 51.0% girls) participated in the first follow-up assessment (T2), which was held two years after T1. Mean age at T2 was 13.56 years (SD = 0.53). The response at T3 was 81.4% (N = 1816, 52.3% girls); mean age was 16.27 years (SD = 0.73).

### Measures

*Emotional and behavioural problem score* was the primary outcome measure. Parent-reported emotional and behavioural problems were assessed by the Child Behaviour Checklist (CBCL) [13] which parents filled in at home. Self-reported emotional and behavioural problems were assessed with the Youth Self Report (YSR) [14], which was filled in at school under supervision of one or more TRAILS assistants. Both questionnaires contain a list of 112 emotional and behavioural problems which can be rated as 0 = not true, 1 = somewhat or sometimes true and 2 = often or very true in the past six months. Total problem scores were derived by averaging the scores on all items.

The main predictor variable was *mental health service (MHS)* use. Parents were asked to report whether they had ever (T1), during the past year (T1), or during the past two years (T2 and T3) visited any MHS for emotional or behavioural problems of their child, and if they had, whether they had also visited this service in the past six months. MHS included child and adolescent inpatient and outpatient services, psychiatrists or psychologists in private practice, community (social) services, psychiatric emergency care, and youth protection services. Scores were dichotomised into having visited at least one MHS or not. Separate scores were made for use prior to T1 and for use in the past six months. Data on MHS use were available for 1885 respondents at T2 and 1464 at T3.

Additional measures included: *temperament*, which was measured during T1 by the parent version of the Early Adolescent Temperament Questionnaire-Revised (EATQ-R) [15]. The EATQ-R is a 62-item questionnaire containing eight domains: Effortful control, Affiliation, Fearfulness, Frustration, Surgency, Shyness, Aggression and Depressed Mood. Temperament is considered a multi-dimensional concept in which low scores on effortful control and affiliation as well as high scores on frustration and fear are associated with emotional and behavioural problems, whereas high scores on effortful control and affiliation have been shown to protect against these problems [16]–[18].

*Preschool behaviour* was assessed retrospectively during T1 with a questionnaire developed for TRAILS, on preschool (age 4–5) child characteristics. The questionnaire contained 17 behavioural, emotional and motor items that parents rate on a five-point scale in relation to their child's peers. Examples are: ‘Was your child bossy, compared to other children?’ and ‘Was your child anxious, compared to other children?’. Factor analysis yielded five dimensions: Anxiety, Motor Behaviour, Aggression, Social Behaviour, and Concentration [19].

*Parental psychopathology* was measured during T1 with the Brief TRAILS Family History Interview, which was administered at home with one of the parents. The questionnaire covered several domains of psychopathology: depression, anxiety, substance abuse, antisocial behaviour and psychosis. The syndromes were introduced by a vignette describing the main DSM-IV characteristics of the disorder, followed by a series of questions assessing lifetime occurrence, professional treatment and medication use. Prevalence rates were comparable to CIDI DSM-IV rates found by direct interviewing in a large population survey [20]. The scores for substance abuse and antisocial behaviour were used to construct a familial vulnerability index for behavioural disorder. The scores for depression and anxiety were used to construct a vulnerability index for emotional disorder [17].

*Socioeconomic position (SEP)* was constructed based on the educational and occupational level of both parents and family income level during T1. Educational level of parents was classified in five categories; occupational level was based on the International Standard Classification for Occupations [21]. Parents reported on the family income. SEP was constructed as the average of the five items, standardised. The SEP scale captured 61.2% of the variance in the five items and had an internal consistency of 0.84.

*Intelligence Quotient (IQ)* was estimated during T1 using the Vocabulary and Block Design subtests from the Revised Wechsler Intelligence Scales for Children [22].

*Stressful life events* were assessed during T2, using a list of 25 life events of which respondents rated whether the event had happened since T1 and how unpleasant it had been. Events were summed to create an overall stress score for the period between T1 and T2, excluding those rated as ‘not unpleasant at all’. At T3 the Event History Calendar (EHC, cf. Caspi *et al* [23]) was administered. The EHC is an interview on important life events, either stressful or pleasant, during the past five years. Both instruments included life events such as death of close relatives, parental divorce, romantic breakup, loss of important friendship and bullying. In addition, the EHC included events such truancy and conflicts between family members. Stressful life events that had occurred between the second and third wave were summed to create an overall stress score during this period.

*Self-esteem* was measured with the Self-Perception Profile for Children (SPPC) [24] during T1. The SPPC evaluates self-esteem in five domains: scholastic competence, social acceptance, athletic competence, physical appearance, and behavioural conduct, as well as global self-worth. Research in a large sample of Dutch adolescents has confirmed the factor structure of the five domains. The questionnaire showed good psychometric properties [25].

*Social skills* were assessed with the Social Skills Rating System (SSRS) during T1. The SSRS is a multi-rater social behaviour assessment package with separate rating forms for teachers and parents [26]. Both teacher and parent forms contain three subscales: Cooperation, Assertion and Self Control. The parent version contains an additional Responsibility subscale. Psychometric properties of the SSRS are satisfactory [26]; an earlier TRAILS study with the SSRS has confirmed its reliability in the current sample [27].

*Peer Acceptance and Rejection* was assessed with peer nominations during T1 [28]. Children were asked which classmates they liked and disliked, for which they could nominate an unlimited number of same-gender and cross-gender classmates. The nominations received for being liked and being disliked were divided by the total number of classmates, that is, the maximum number of possible nominations. This way, the scores were transformed into proportions meaning that differences in class-size are taken into account. Scores for peer acceptance (like) and peer rejection (dislike) thus ranged from 0 to 1 [29].

*Perceived parenting* was assessed during T1 with the the Egna Minnen Beträffande Uppfostran (My Memories of Upbringing) for Children (EMBU-C). We used the shortened version [30], of which the psychometric properties are satisfactory [30], [31]. The EMCU-C contains three subscales: emotional warmth, rejection and overprotection. The scale Emotional Warmth is characterised by giving special attention, praising for approved behaviour, unconditional love, and being supportive and affectionately demonstrative (“Do your parents make it obvious that they love you?”). Rejection is characterised by hostility, punishment, derogation and blaming of child (“Do your parents sometimes punish you even though you have done nothing wrong”). Overprotection is characterised by fearfulness and anxiety for the child's safety, guilt engendering, and intrusiveness (“Do you feel that your parents are extremely anxious that something will happen to you?”). The answers for both parents were highly correlated, so we combined them into a single score [32].

### Statistical analysis

In mental health care research, randomized controlled trials (RCTs) are considered the golden standard when studying the effectiveness of treatment. Randomization minimizes pretreatment differences between the experimental groups, so that any posttreatment differences can be assumed to be due to the treatment condition. However, randomization is not always possible or desirable [33], [34] and RCTs are often conducted in highly selective patient samples, which threatens the external validity of these studies [35]. Observational studies therefore offer valuable complementary information about treatment outcomes. Since observational studies run the risk of (unmeasured) pretreatment differences between the intervention and control group, a phenomenon often referred to as confounding by indication, specific statistical techniques need to be applied to be able to draw valid conclusions about treatment effectiveness [36]. Three of these techniques, which are described below, were used in the current study on differences in the naturalistic course of emotional and behavioural problems between adolescents who had received mental health treatment and those who had not.

#### 1. Adjusting for possible confounders

We first performed a multivariate linear regression analysis with MHS use as primary predictor and emotional and behavioural problems at follow up as outcome variables, adjusting for a wide range of potential confounders. In order to be a confounder, a variable should be associated with both the predictor (receiving treatment) and the outcome (follow-up problem score). In our analyses, the most important confounder was pretreatment severity of emotional and behavioural problems. In addition, we selected various other vulnerability markers (i.e., variables assumed to increase both the likelihood of treatment and mental health problems) as putative confounders, as well as a number of resilience markers, which might protect against treatment and mental health problems. Vulnerability markers included an unfavourable temperament (low scores on effortful control and affiliation, high on fearfulness and aggression [16]), difficult preschool behaviour (high scores on aggression, low on social behaviour [19]), low IQ [22], low SEP [37], parental rejection or overprotection [32], mental health care use prior to T1, parental emotional or behavioural disorders [17], poor social skills [27], and peer rejection [29]. Selected resilience markers were a favourable temperament (high scores on effortful control and affiliation, low on fearfulness and aggression [16]), self-esteem, parental warmth [38], and peer acceptance [32]. A final putative confounder was exposure to stressful life events [39]. The effect of all putative confounders on mental health problems and mental health care use was tested univariately. When statistically significant (p<0.05) the variable was included in the multivariate linear regression analysis. In the multivariate linear regression analysis, we adopted a stepwise approach to predict mental health problem scores at follow up. First, we included only MHS use (the predictor of interest) and baseline severity of emotional and behavioural problems. In the second step, we additionally included all other vulnerability and resilience markers that had been shown to be related to mental health problems and mental health care use (see above). Third, we added stressful life events.

#### 2. Propensity score matching

The second technique used to prevent confounding by indication is called propensity score matching [36], [40], [41]. This method has been used in various fields, among which medicine [42], [43], social sciences [44] and mental health care research [45]. In this approach, treated cases are matched to control cases based on a so-called propensity score, that is, the likelihood of being assigned treatment, given a set of pre-treatment observed characteristics [41]. Propensity score matching thus mimics a randomized control trial, although *un*observed differences between cases and controls are not accounted for. The propensity score can be derived from a logistic regression analysis in which treatment is predicted by a set of preselected variables known to influence help-seeking and service use [2]. A person's propensity score is the predicted treatment probability, which can range from 0 (lowest probability) to 1(highest probability). Please note that the propensity score reflects a probability (i.e., having characteristics generally associated with treatment), not actual service use: theoretically, a respondent could have a propensity score of .99 but not have received treatment.

The variables that were selected to derive the propensity score in the present study are presented in Appendix 1. After having derived the propensity scores, treated adolescents were matched to non-treated adolescents with a comparable (the same or nearest by) propensity score. Hence, this approach implied a reduction of the dataset because we used only controls (i.e., untreated adolescents) that could be matched to a treated adolescent. In total, 188 adolescents used MHS, of whom 11% (N = 21) could not be matched to a control with a comparable propensity score. These cases were excluded, leaving 167 MHS users and 167 MHS non-users, assumed to be comparable with respect to the likelihood of receiving treatment for emotional and behavioural problems. All covariates were equally distributed across the two groups, indicating that they were comparable in all respects, except for treatment condition. These two groups were compared with regard to the course of their emotional and behavioural problems.

Adolescents were considered ‘controls’ if they did not receive treatment between T1 and T2. Some of the controls, however, did receive treatment two-to-four years later, between T2 and T3. Likewise, some cases received treatment only between T1 and T2, others also between T2 and T3. To examine treatment effects throughout the three measurement waves, we divided the treated adolescents and their matched controls each into two subcategories, yielding four groups. The first group (N = 146) consisted of controls who did not receive treatment between T2 and T3 either, hence did not use MHS throughout the waves. The second group (N = 114) consisted of adolescents who received treatment between T1 and T2, but did not between T2 and T3. The third group (N = 21) consisted of controls who started using MHS after T2, and the fourth group (N = 53) of treated adolescents who continued to receive treatment between T2 and T3. These four groups were compared with regard to their course of emotional and behavioural problems throughout the waves.

#### 3. Sensitivity analyses

The third approach to enhance the validity of the conclusions consisted of sensitivity analyses to examine the robustness of the findings. We conducted four robustness checks. First, we used both parent- and self-reported emotional and behavioural problems to reduce informant bias. Second, we performed the multivariate linear regression analysis (approach 1) with regard to not only MHS use between T1 and T2, but also MHS use between T2 and T3. Third, because mental health care use can concern various types of care, we repeated the linear multivariate regression analysis for clinical (24 hours) mental health care and outpatient mental health care separately. Fourth, to investigate possible bias because of selective dropout, we compared the results with those after multiple imputation of missing data. Missing data were estimated by linear regression analysis using all relevant observations. Five new datasets were created, which were used for the analyses described above. Estimates from all datasets were then pooled, using Rubin's rules [46]

## Results

### Descriptive statistics

Adolescents who used MHS were, on average, more often male, had higher total problem scores on the CBCL and the YSR, had higher familial loadings on both internalizing and externalizing disorders, and had a lower IQ than those who did not. Of the T1 respondents who scored above the 85^th^ percentile of the CBCL, which is considered a clinical cut-off, 38% visited MHS services, versus 3% of the respondents with the lowest (<P25) CBCL scores.

### Results approach 1

Table 1 shows the regression coefficients of the putative predictors of parent-reported problems at T2, tested univariately. Results of the univariate regression analysis predicting mental health care use (available upon request) show statistically significant associations between all putative predictors and mental health care use, except for SEP which was marginally significant. The associations were in the same direction as the associations presented in Table 1, with baseline parent-reported emotional and behavioural problem scores and previous mental health care use being important predictors, and a favourable temperament, social skills and emotional warmth of parents protecting against both emotional and behavioural problems at follow up as well as against mental health care use.

**Table 1:** Univariate regression analyses with CBCL scores at T2 as dependent variable (standardised regression coefficients with standard errors).

		ß (SE)
CBCL score T1		0.68 (0.02)***
MHS use between T1 and T2		0.38 (0.02)***
Gender (male)		0.04 (0.02)
IQ		−0.14 (0.02)***
Socioeconomic position		−0.16 (0.02)***
Familial vulnerability behavioural disorder		0.16 (0.02)***
Familial vulnerability emotional disorder		0.23 (0.02)***
Temperament:	Effortful control	−0.43 (0.02)***
	Affiliation	−0.09 (0.02)***
	Fearfulness	0.24 (0.02)***
	Frustration	0.42 (0.02)***
	Surgency	−0.07 (0.02)**
	Shyness	0.05 (0.02)**
	Aggression	0.41 (0.02)***
	Depressed mood	0.42 (0.02)***
Preschool Behaviour:	Anxiety	0.16 (0.02)***
	Motor Behaviour	−0.14 (0.02)***
	Aggression	0.25 (0.02)***
	Social Behaviour	−0.13 (0.02)***
	Concentration	−0.29 (0.02)***
Previous MHS use		0.33 (0.02)***
Self-esteem:	Learning	−0.06 (0.03)**
	Friends	−0.16 (0.03)***
	Sport	−0.03 (0.02)
	Appearance	0.07 (0.03)*
	Behaviour	−0.13 (0.03)***
	General	−0.20 (0.03)**
Social skills:	Cooperation (t)	−0.07 (0.03)*
	Assertion (t)	−0.02 (0.03)
	Self-control (t)	−0.07 (0.04)
	Cooperation (p)	−0.11 (0.03)***
	Responsibility (p)	0.15 (0.03)***
	Assertion (p)	−0.18 (0.03)***
	Self-control (p)	−0.34 (0.03)***
Peer acceptance		−0.15 (0.02)***
Peer rejection		0.16 (0.03)***
Emotional warmth of parents		−0.13 (0.03)***
Parental overprotection		0.13 (0.02)***
Parental rejection		0.21 (0.02)***
Life events past two years		0.22 (0.02)***

CBCL, Child Behaviour Checklist; MHS, Mental Health Services; IQ, Intelligence Quotient; EXT, Externalising disorder; INT, internalising disorder; (t) teacher and (p) parent rating.

* p-value<0.05;

** p-value<0.01;

*** p-value<0.001.

Table 2 shows the result of the multivariate linear regression model with MHS use as predictor of problems at follow up, adjusted for T1 severity, markers of adolescent vulnerability and resilience, and life events between the assessment waves.

**Table 2:** CBCL scores at T2 predicted by MHS use between T1 and T2, adjusted for baseline severity of symptoms (model 1); baseline severity and markers of adolescents vulnerability and resilience (model 2); and baseline severity, markers of adolescent vulnerability and resilience, and stressful life events (model 3).

		Model 1	Model 2	Model 3
		ß (SE)	ß (SE)	ß (SE)
MHS use between T1 and T2		0.21 (0.02)***	0.21 (0.03)***	0.20 (0.03)***
Total problem scores (CBCL) T1		0.62 (0.02)***	0.48 (0.04)***	0.48 (0.04)***
Gender (male)			−0.03 (0.03)	−0.01 (0.03)
IQ			−0.03 (0.03)	−0.02 (0.03)
Socioecomic position			−0.05 (0.03)	−0.04 (0.03)
Familial vulnerability behavioural disorder			0.02 (0.03)	0.01 (0.03)
Familial vulnerability emotional disorder			0.10 (0.03)***	0.11 (0.03)***
Temperament:	Effortful control		−0.06 (0.04)	−0.06 (0.04)
	Affiliation		−0.01 (0.03)	−0.01 (0.04)
	Fear		−0.02 (0.03)	−0.00 (0.03)
	Frustration		−0.03 (0.04)	−0.03 (0.04)
	Surgency		−0.00 (0.03)	−0.01 (0.03)
	Shyness		−0.01 (0.04)	−0.01 (0.04)
	Aggression		0.09 (0.04)*	0.07 (0.04)
	Depressed mood		0.00 (0.04)	−0.00 (0.04)
Preschool Behaviour	Anxiety		−0.05 (0.04)	−0.06 (0.04)
	Motor Behaviour		−0.06 (0.03)	−0.07 (0.03)*
	Aggression		0.06 (0.04)	0.07 (0.04)
	Social Behaviour		0.03 (0.03)	0.02 (0.03)
	Concentration		0.03 (0.04)	0.04 (0.04)
Previous MHS use			0.01 (0.03)	−0.00 (0.03)
Self-esteem:	Learning		−0.00 (0.04)	−0.01 (0.04)
	Friends		−0.05 (0.04)	−0.04 (0.04)
	Sport		−0.04 (0.03)	−0.04 (0.03)
	Appearance		−0.03 (0.04)	−0.01 (0.04)
	Behaviour		−0.04 (0.03)	−0.05 (0.03)
	General		0.05 (0.05)	0.04 (0.05)
Social skills:	Cooperation (t)		0.03 (0.04)	0.06 (0.04)
	Assertion (t)		−0.01 (0.04)	−0.01 (0.04)
	Self-control (t)		0.02 (0.04)	−0.00 (0.05)
	Cooperation (p)		−0.03 (0.04)	−0.04 (0.04)
	Responsibility (p)		0.05 (0.04)	0.05 (0.04)
	Assertion (p)		−0.01 (0.04)	−0.01 (0.04)
	Self-control (p)		−0.01(0.04)	−0.03 (0.04)
Peer acceptance			0.00 (0.03)	−0.01 (0.03)
Peer rejection			0.01 (0.03)	−0.00 (0.03)
Emotional warmth of parents			−0.00 (0.03)	−0.01 (0.03)
Parental overprotection			0.01 (0.03)	−0.00 (0.03)
Parental rejection			0.05 (0.04)	0.04 (0.04)
Life events past two years				0.14 (0.03)

Standardised regression coefficients (ß) and standard errors (SE) are presented.

CBCL, Child Behaviour Checklist; MHS, Mental Health Services; IQ, Intelligence Quotient; (t) teacher and (p) parent rating. Adjusted R^2^ model 1: 0.51; model 2: 0.52; model 3: 0.52.

* p-value<0.05;

** p-value<0.01;

*** p-value<0.001.

MHS use predicted increased total problems as reported by parents at T2, adjusted for severity of symptoms at T1. The association between MHS use and T2 problems was not confounded by any of the before-mentioned risk or protective factors (Table 2).

### Results approach 2

**Figure 1:**
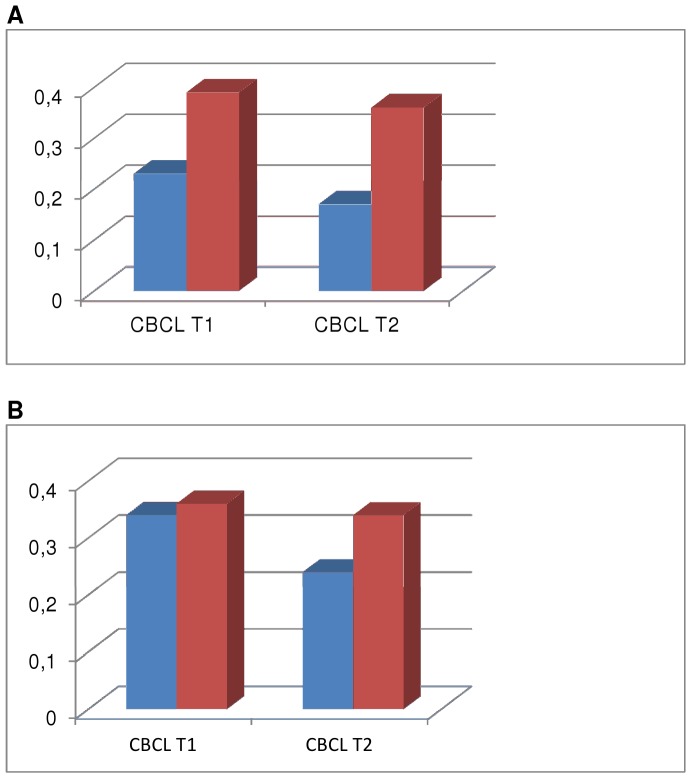
A and B. Uncorrected and propensity adjusted mean CBCL-scores of TRAILS participants with and without MHS use. In figure 1A, mean total problem scores (CBCL) are displayed of TRAILS participants with and without MHS use at baseline (T1) and follow up (T2). In figure 1B, mean total problem scores (CBCL) are displayed of propensity matched TRAILS participants with and without MHS use. The participants who did not use MHS had, at baseline, the same propensity (i.e. likelihood) to receive MHS as the participants who actually used MHS. Legend A: Red square denotes TRAILS participants with MHS use (N = 188). Blue square denotes TRAILS participants without MHS use (N = 1692). CBCL: Child Behaviour Checklist, total problem score. MHS: Mental health services. Legend B: Red square denotes TRAILS participants with MHS use (N = 167). Blue square denotes propensity score matched TRAILS participants without MHS use (N = 167). CBCL: Child Behaviour Checklist, total problem score. MHS: Mental health services.

Figure 1 shows the differences between uncorrected and propensity-adjusted mean CBCL scores at T1 and T2 of TRAILS participants with and without MHS use. As can be seen in Figure 1a, MHS users had high initial problem scores which had only marginally decreased at T2, whereas non-users have lower initial scores which had decreased more at T2. Figure 1b shows the results based on the propensity score matching. The initial CBCL scores of MHS users and non-users were approximately the same (non-significant difference), but the scores of non-users had decreased remarkably at T2 while they continued to be high in the MHS users.

**Figure 2:**
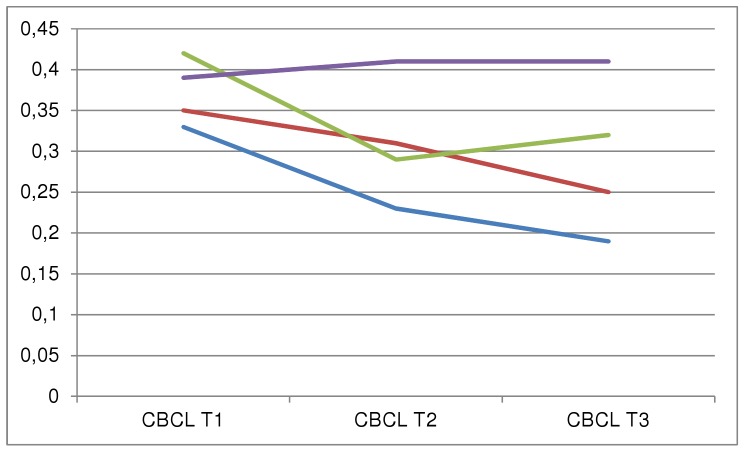
MHS use and CBCL scores across the three measurement waves. In this figure, mean CBCL total problem scores are displayed of propensity matched TRAILS participants that did or did not use MHS during a certain time period. Legend: Blue line denotes TRAILS participants with no MHS at any time (N = 146). Red line denotes TRAILS participants with MHS between T1 and T2 (N = 114). Green line denotes TRAILS participants with MHS between T2 and T3 (N = 21). Purple line denotes TRAILS participants with continuous MHS use (N = 53). CBCL: Child Behaviour Checklist, total problem score. MHS: Mental health services.

Figure 2 displays the problem scores across the three waves after having divided the matched initial MHS users and non-users into four groups, based on further MHS use. Future MHS users, as well as initial users who continued to use MHS, appeared to have higher initial (T1) CBCL scores than non-users and participants with MHS use between T1 and T2 only. Furthermore, adolescents who never used MHS but had equal propensity scores as the MHS users showed the largest reduction in emotional and behavioural problems over time (mean reduction from T1–T3 0.11, t = 6.327, df 87, p-value<0.001). Adolescents who had accessed MHS only between T1 and T2 started with slightly higher problem scores which also decreased in time (mean reduction from T1–T3 0.09, t = 4.6, df 66, p-value<0.001). Adolescents who had accessed MHS only between T2 and T3 showed a significant reduction in problem score before they started using MHS (mean reduction 0.19, t = 4.54, df 20, p-value<0.001), after which their problem scores increased, although this change was not statistically significant. Adolescents who used MHS continuously, i.e. between T1 and T2 as well as between T2 and T3, stayed on a high problem level throughout the three waves, with no significant increase or decrease in problem levels.

### Results approach 3

Repeating the multivariable regression analysis with self-reported problem score as outcome measure showed comparable results. Likewise, using data from the T2–T3 time period did not alter the results, neither for parent-reported nor for self-reported problem scores as outcome measure. When we used only clinical mental health care (and covariates) as predictor of parent-reported problem scores, the effect size became smaller and non-significant (β 0.03, SE 0.13, n.s.). With outpatient mental health care only, the effect size increased and remained statistically significant (β 0.26, SE 0.12, p-value = 0.027). For mental health care use between T2 and T3, the effect sizes decreased for both clinical care (β 0.13) and outpatient care (β 0.03), and none of the effects reached statistical significance. The multivariate regression analysis with self-reported problems as outcome measure yielded similar results for clinical and outpatient care separately as for total MHS use for the T1–T2 period. For the T2–T3 time period, the effect of clinical mental health care on self-reported problems was not significant (β −0.09, SE 0.14, n.s.), while for outpatient care, the effect increased (β 0.36, SE 0.14, p-value = 0.013).

Using self-reported emotional and behavioural problems as outcome measure in the propensity score matching approach yielded slightly deviating results for the group of adolescents that used MHS continuously. Their problem score first increased during T2, then decreased during T3, when it fell slightly below the T1 level. This effect is not seen in the parent-reported problem score for this group. The course of problems for all other groups was similar to what was found with parent-reported problems as outcome measure. The dataset with imputed missing values yielded equivalent results as the ones presented here. More details on any of these analyses are available upon request.

## Discussion

### Main findings

In our general population study, adolescents who used MHS had more emotional and behavioural problems than those who did not use MHS, as might be expected. However, MHS use also predicted high problem scores at follow up, and this association was not confounded by any measured marker of adolescent vulnerability or resilience, nor by baseline problem severity. The results were regardless of the informant (i.e. parent- or self-reported problems). Regarding the type of mental health care (clinical or outpatient), the results seem to indicate that these effects are more pronounced for outpatient care than for clinical care, however, in both cases, there is no evidence that the problem scores of the treated group improved more at follow-up than the problem scores of the non-treated group. The propensity score method enabled us to compare the course of mental health problems of a group of treatment users and non-users with comparable likelihood to receive treatment at baseline. The results showed that follow-up problem scores decreased for both non-users and short-term users, but more strongly for non-users. Problem scores of adolescents who use MHS persistently across all waves did not decrease at all.

### Strengths and weaknesses

Studying the outcome of interventions in a naturalistic setting is hampered by the absence of randomisation. The propensity score matching method compensates for that omission by comparing individuals with an equal likelihood of accessing health care services, based on a number of factors that have been shown to influence help seeking behaviour [2], [5]. The matching allowed us to compare the course of emotional and behavioural problems of adolescents with and without MHS use who were, at baseline, equally likely to access MHS based on their propensity score. As opposed to RCTs, which include only adolescents meeting certain inclusion criteria, our respondents came from the general population, reflecting all naturally occurring co-morbidity patterns. This increases the external validity of the results. The propensity score method has proven to be a valuable method to assess treatment effectiveness when randomisation is not possible, or desirable, such as in the situation where one wants to compare the course of problems of treatment users versus non-users. The propensity score method allowed us to balance all measured covariates equally among users and non-users. The large amount of data collected in the TRAILS cohort made it possible to control for a wide range of possible confounders, among which markers of adolescent vulnerability and resilience. The multiple assessment waves enabled the comparison of outcomes over 6 years, with interventions starting and ending at different time points. The multi-informant character of the TRAILS cohort enabled us to use both parent-reported and child-reported measures.

The findings and interpretations as presented in this paper should be regarded in the context of a number of limitations. First, although we adjusted for a wide range of confounders in the multivariate regression analyses and used an equally wide range of variables to calculate the propensity scores, the outcome may have been influenced by variables that were unobserved or difficult to measure. Possible unmeasured differences between treated and untreated adolescents could be, for instance, the clinical view of gatekeepers deciding whether or not to refer adolescents to specialist care, the (perceived) distance to specialist mental health services, (un)favourable past experiences with mental health services, or social support. We did adjust for socioeconomic position, parental education and parental psychopathology, which probably encompass at least part of the bias due to these factors, but may not have been able to exclude all possible bias. Second, information on MHS use was limited in our study: we know which mental health services were visited, but have no information on the duration or intensity of the treatment. Like in regular clinical practice, participants received a variety of specialist mental health care services. We should like to emphasise that, although for research purposes this heterogeneity might be a limitation, an overall (beneficial) effect of MHS use seems absent.

### Interpretation of the findings

Confounding by indication, i.e. prognostic factors influencing treatment choices as well as the outcome of treatment, cannot be completely excluded, even though we used sophisticated methods in trying to control for it. However, it might be worthwhile to explore alternative explanations of the findings. For instance, Weisz *et al.* [8] may have been right in signalling that ‘care as usual’ appears to lack effectiveness. This lack of effect may be caused by the diverse, complex reality of everyday settings, compared to the controlled situation of RCTs. It is possible that evidence-based treatments are not implemented or are implemented for the wrong target group. Alternatively, evidence-based treatments may not be feasible in everyday settings due to a shortage or inadequate schooling of staff [47].

Another possible explanation may be that adolescents using MHS have parents that are more concerned or more troubled by the behaviour of their child compared to parents of adolescents without MHS use. These worries may result in a stronger tendency to access MHS as well as in more severe ratings of their child's behaviour. These speculations are supported by the higher overall explained variance of the model where MHS use is associated with parent-reported problem scores (R^2^ = 0.52) than of the model predicting self-reported problem scores (R^2^ = 0.46). On the other hand, use of self-reported problem scores still resulted in a positive association with MHS use, so mono-informant bias is unlikely to explain the association between MHS use and mental health problems completely.

A final explanation for our findings may be sought in unmeasured variance in the duration of treatment. Angold *et al.* [48] have shown a dose-response relation with regard to the effectiveness of MHS. Adjusted for severity of problems, they showed that psychiatric symptoms started to decrease only after nine therapy sessions. Possibly, our sample contained a group of adolescents with high MHS needs who dropped out of treatment too soon. In a study by Laratatou *et al*, for instance, approximately 60% of children and adolescents did not comply with treatment, with almost half of them leaving after the first appointment [49]. Early treatment dropout may worsen problem scores. On the other hand, adolescents with relatively mild symptoms who stay in treatment too long may have increasing problems as well, because mental health care visits can lead to (self) stigmatisation [50]. MHS use may support adolescents in dealing with emotional and behavioural problems but also give them the impression that they are needy and weak, incapable of solving their own problems. Unfortunately, we did not collect detailed information on the duration of the MHS use in our study, but future studies should investigate whether these factors possibly increase emotional and behavioural problems rather than decrease them.

### Conclusion

Although residual confounding by indication cannot be excluded, our findings urge for a critical analysis of treatment practices in everyday settings. Are evidence-based treatments implemented, and, if they are not, what are the obstacles? If they are implemented, are they effective in real world settings? Is it possible that MHS have adverse effects for some adolescents, and if so, who are these adolescents? However, before such implications are considered, replication studies are necessary to reveal whether or not an artefact is responsible for the results.
